# Immune Response to *Salmonella* Enteritidis Infection in Broilers Immunized Orally With Chitosan-Based *Salmonella* Subunit Nanoparticle Vaccine

**DOI:** 10.3389/fimmu.2020.00935

**Published:** 2020-05-19

**Authors:** Yi Han, Sankar Renu, Veerupaxagouda Patil, Jennifer Schrock, Ninoshkaly Feliciano-Ruiz, Ramesh Selvaraj, Gourapura J. Renukaradhya

**Affiliations:** ^1^Food Animal Health Research Program, Ohio Agricultural Research and Development Center, Wooster, OH, United States; ^2^Department of Veterinary Preventive Medicine, College of Veterinary Medicine, The Ohio State University, Columbus, OH, United States; ^3^Department of Poultry Science, University of Georgia, Athens, GA, United States

**Keywords:** broilers, *Salmonella* enteritidis, chitosan nanoparticle vaccine, immune response, protection

## Abstract

*Salmonella enterica* serovar Enteritidis (*S*. Enteritidis, SE) infection in broilers causes a huge economic loss and public health risk. We previously demonstrated that orally delivered chitosan based (CS) *Salmonella* subunit nanoparticle (NP) vaccine containing immunogenic outer membrane proteins (OMP) and flagellin (FLA) of SE [CS-NP(OMP+FLA)] induces immune response in broilers. The objective of this study was to evaluate the dose- and age-dependent response and efficacy of CS-NP(OMP+FLA) vaccine in broilers. Three-day old birds were vaccinated and boosted once or twice. Additional groups were vaccinated at three weeks with no booster or boosted once a week later. Each dose of CS-NP vaccine had either 10 or 50 μg of OMP+FLA antigens. Our data revealed that two doses of vaccine were required to induce substantial immune response. Birds received 2 doses of CS-NP(OMP+FLA) vaccine at 3 days and 3 weeks of age with 10 μg antigens, and birds inoculated twice at 3 and 4 weeks of age with 50 μg antigens had lowest challenged bacterial load in the cecal contents with over 0.5 log_10_ reduction. In CS-NP(OMP+FLA) vaccinated birds, antigen-specific splenocyte proliferation, mucosal and systemic antibody response and the frequency of IFNγ-producing T cells were increased compared to control groups. At the molecular level, in the cecal tonsils of CS-NP(OMP+FLA) immunized birds, mRNA levels of toll-like receptor (TLR) 2 and TLR 4, and cytokines IL-4 and IL-10 were upregulated. The CS-NP(OMP+FLA) vaccine given orally has the potential to induce a protective immune response against SE infection in broilers.

## Introduction

Salmonellosis is responsible for ~1 million foodborne illnesses, 20,000 hospitalizations and 4,380 deaths annually in the United States (1). *Salmonella* spp. have been detected in 20% of broiler chickens and 44.6% of ground chicken meat (2). Among the 2,600 *Salmonella* serotypes, *Salmonella* Enteritidis (SE) is the predominant serotype associated with human disease in most countries (3). In

2013, the Food Safety and Inspection Service (FSIS) of USDA released the *Salmonella* Action Plan to address the threat of *Salmonella* in poultry products. Chicken immune organs begin developing during early embryogenesis and are fully functional by 2–3 weeks after hatching. Vaccination against *Salmonella* is considered a viable control strategy. During our market validation of *Salmonella* vaccination in poultry under a I-Corps^@^Ohio activity (4), we interviewed 67 people throughout US comprising of poultry veterinarians, research scientists, consultants, laboratory diagnosticians, farm managers, vaccine manufacturers and USDA regulators of poultry products. The survey revealed that ~1% of broilers receive live *Salmonella* vaccine within first week of hatching, and not anytime later due to risk of vaccine bacteria getting into human food chain. However, most of the broiler breeders are vaccinated with live and killed *Salmonella* vaccines. This approach is expected to confer maternal immunity in chicks. But none of the current vaccination methods provide satisfactory control of *Salmonella* in broilers.

Our previous study in broilers using chitosan nanoparticle (CS-NP)-entrapped with SE outer membrane proteins (OMP) and flagellin (FLA) with surface conjugated FLA [CS-NP(OMP+FLA)] delivered orally induced innate toll-like receptors (TLR) expression and antigen specific lymphocyte proliferation responses (Yi and Renukaradhya, manuscript submitted). In layers vaccinated orally, CS-NP(OMP+FLA) induced cell mediated and humoral immune responses (5). The CS-NP(OMP+FLA) particles were around 500 nm diameter suitable for efficient uptake by antigen presenting cells (6). The goal of this study is to evaluate the dose- and age-dependent response and efficacy of CS-NP(OMP+FLA) vaccine in broilers. Our hypothesis is that the candidate nanovaccine induces both antibody and cell mediated immune response and reduces bacterial colonization in the intestines of broilers. In this study, we evaluated the efficacy of CS-NP(OMP+FLA) in broilers vaccinated orally at two different ages and using two different doses of vaccine antigens.

## Materials and Methods

### Experimental Animals, Bacteria, and Vaccine

Day-old Cornish Cross breed broilers were purchased from a commercial hatchery (Ashland, OH, USA). Birds were confirmed *Salmonella* free upon arrival by plating the cloacal swab samples on Xylose Lysine Deoxycholate (XLD, Sigma-Aldrich, St Louis, MO, USA) agar plates. The chickens were reared on flooring with pine shavings as litter in an environmentally controlled BSL2 animal facility; lighting was provided 18 h/day. Birds were fed with mash corn-soybean diet free from antibiotics. Feed and water were provided *ad-libitum*.

The poultry isolate of SE, bacteriophage type 13A (7), was originally obtained from the USDA National Veterinary Services Laboratory (Ames, IA, USA). Killed SE whole protein antigen (KAg), OMP and FLA protein were harvested as previously described with modifications (5, 8–10). Chitosan nanoparticle-based *Salmonella* subunit vaccine was prepared by an ionic gelation method and characterized as previously described (5). In each dose of CS-NP(OMP+FLA) vaccine, equal amount (5 or 25 μg) of OMP and FLA were entrapped in CS-NP.

### Experimental Design

On the day of hatch, 68 *Salmonella*-free broilers were received from a commercial hatchery. Birds were randomly grouped into 12 groups (*n* = 5 or 6 birds per group). Groups of chicks received two different vaccine doses with prime-vaccination at either 3-day or 3-weeks of age (Table 1). A control soluble antigen group (50 μg antigens/bird) Sol.Ag (OMP+FLA) was included. Birds received prime vaccination at 3 days of age had either received a total of 2 or 3 doses, whereas the groups received the prime vaccination at 3-weeks of age received either 1 or 2 doses of vaccine. Each vaccine dose had OMP+FLA of either 10 or 50 μg entrapped in CS NPs. At 5-weeks of age, all birds except one of the two mock control (unvaccinated) group were challenged orally with pre-titrated dose of SE 5 ×10^8^ CFU/bird. Ten days after challenge, all birds were euthanized and samples of blood, cloacal swab, bile, small intestine, spleen, cecal tonsils and cecal contents were collected from each bird. The aliquots of serum, cloacal swab fluid, small intestine wash fluid and bile samples were stored at −20°C until used in antibody analysis. Spleens were collected for use in splenocytes proliferation assay and the frequency of IFNγ-producing T cells was determined by flow cytometry. Bacterial shedding results were detected by plating cecal contents on nalidixic acid resistant XLD agar plates.

**Table 1:** Experimental animal groups.

**Group#**	**Vaccine received**	**N**	**1st dose/age**	**2nd dose/age**	**3rd dose/age**	**Challenged/age**	**Total dose#**
1	CS-NP(OMP+FLA) 50 μg	6	3 day	3 week	NA	5 week	2
2	CS-NP(OMP+FLA) 50 μg	6	3 day	3 week	4 week	5 week	3
3	CS-NP(OMP+FLA) 10 μg	6	3 day	3 week	NA	5 week	2
4	CS-NP(OMP+FLA) 10 μg	6	3 day	3 week	4 week	5 week	3
5	Sol. Ag(OMP+FLA) 50 μg	5	3 day	3 week	4 week	5 week	3
6	CS-NP(OMP+FLA) 50 μg	6	3 week	NA	NA	5 week	1
7	CS-NP(OMP+FLA) 50 μg	6	3 week	4 week	NA	5 week	2
8	CS-NP(OMP+FLA) 10 μg	6	3 week	NA	NA	5 week	1
9	CS-NP(OMP+FLA) 10 μg	6	3 week	4 week	NA	5 week	2
10	Sol. Ag(OMP+FLA) 50 μg	5	3 week	4 week	NA	5 week	2
11	PBS	5	3 day	3 week	4 week	NA	3
12	PBS	5	3 day	3 week	4 week	5 week	3

*NA, Not applicable; N, Sample size*.

### Enzyme-Linked Immunosorbent Assay (ELISA)

The procedure for specific isotype antibody detection was performed as described previously (11). Briefly, flat-bottom 96-well plates (Greiner bio-one, Frickenhausen, Germany) were coated with pre-titrated optimal amounts of OMP or FLA proteins (375 ng/well for IgA detection; 50 ng/well for IgG detection) in carbonate-bicarbonate buffer (pH 9.6) overnight at 4°C. Plates were washed three times with PBS containing 0.05% Tween 20 (PBS-T), and non-specific binding sites in the plates were blocked using blocking buffer containing 5% (v/v) skim milk in PBS-T (Nestle, Vevey, Switzerland) for 1 h at 37°C. Sera were diluted 1:800, cloacal swab fluid and small intestine wash were diluted 1:1 and bile samples diluted 1:800 in blocking buffer. Diluted samples were added to marked triplicate wells and incubated at 37°C for 1 h. Plates were washed three times and bound antibodies were detected by treating with goat anti-chicken IgA-HRP 1:3000 or IgY-HRP 1:10,000 (Gallus Immunotech, Shirley, MA, USA) diluted in 2.5% skim milk and incubated at 37°C for 1 h. The plates were washed with PBS-T and 3,3′,5,5′-Tetramethylbenzidine (TMB; SeraCare, Milford, MA, USA) was added and incubated at room temperature in the dark for 10–20 min. The reaction was stopped by adding 1 M phosphoric acid and the optical density (OD) values at 450 nm was determined with a microplate reader (Molecular devices, CA, USA).

### Splenocyte Proliferation Assay

The procedure for identifying the specific lymphocyte proliferation index was performed as described previously (11). Briefly, splenocytes were prepared by passing spleen through a cell strainer diluted in sterile PBS. The resultant cell suspension was added onto the equal volume of Ficoll-paque plus solution. Red blood cells were removed by centrifugation at 450 × g for 30 min at 4°C. The splenocytes in the interface were harvested and adjusted to a concentration of 10^7^ cells/ml in RPMI 1640 media (Gibco-BRL, Paisley, UK), containing 100 U/ml antibiotic-antimycotic and 10% fetal bovine serum. The cells were seeded in 96-well cell culture flat-bottom plates (100 μl/well) in triplicate wells for each animal sample and treated with RPMI 1640 media containing pre-titrated amounts of KAg (10 μg/ml), OMP (10 μg/ml) and FLA (10 μg/ml). Cells were incubated at 39°C in an atmosphere of 5% CO_2_ for 48 h, and then added the CellTiter 96® Aqueous One Solution Reagent (Promega, Madison, WI, USA) and incubated for additional 4 h. Optical density (OD) values were recorded at 490 nm absorbance by a spectrophotometer (Molecular devices, CA, USA). The stimulation index (SI) was calculated from OD value of stimulated cells divided by the value of mock control cells.

### Antigen-Specific Lymphocyte Response by Flow Cytometry Analysis

The frequency of antigen-specific activated lymphocyte subsets were detected as described previously (12) with few modifications. Ten million fresh splenocytes of each bird isolated on the day of necropsy were seeded in a 24-well flat bottom plate in 2 ml enriched RPMI. The cells were stimulated with 10 μg /ml of OMP+FLA mixture for 72 h. Contaminated samples during incubation were removed from the following data analysis. Protein transport inhibitors Brefeldin A (GolgiPlug; BD Bioscience, San Jose, CA, USA Cat#51-2301KZ) and Monensin (GolgiStop; BD Bioscience, CA, USA Cat#51-2092KZ) were added for the last 6 h of incubation. Cells were harvested from the plate, washed, blocked with 1% normal rabbit serum and split into 4 wells in a 96-well round bottom plate. Cells were first surface immunostained for chicken lymphocyte subsets using specific antibodies CD3, CD8α and TCRγδ or the corresponding isotype control antibody tagged with fluorescein or biotin (Table 2). This was followed by fixation using 1% paraformaldehyde and after washing cells were resuspended in FACS buffer. Intracellular IFNγ staining was performed by an indirect method using specific antibodies (Table 2) as described previously (13). Briefly, cells were washed in 1 × PBS and subjected to permeabilization using 10% saponin buffer for 45 min, 22°C. After washing in 0.1% saponin buffer, cells were incubated with 4 μg/ml of anti-chicken IFNγ polyclonal antibody (BioRad, CA, USA) in 50 μl for 45 min, 4°C. Cells were washed once in saponin buffer and treated with Goat anti-Rabbit IgG AF647 secondary antibody (0.1 μg/ml) for 45 min, 4°C. Normal rabbit serum was used as a negative control. Cells were washed and resuspended in 200 μl FACS buffer and transferred to FACS tubes. The samples were acquired using BD FACS Aria II (BD Biosciences, San Jose, CA, USA) and lymphocyte gates were made (**Figure 5**) using the FlowJo software (Tree Star, Ashland, OR, USA). The frequency of specific T-cell subpopulations was calculated as the percent of total CD3^+^ lymphocytes.

**Table 2:** Antibodies used for cell surface and intracellular IFNγ staining.

**Antibody**	**Cat#; company**
Live/dead-FITC	Cat# L34970; ThermoFisher scientific, MA, USA
Mouse anti-chicken CD3 AF700	Cat# 8200-27; SouthernBiotech, birmingham, AL, USA
Mouse anti-chicken CD4 FITC	Cat# 8210-02; SouthernBiotech, birmingham, AL, USA
Mouse anti-chicken CD8α PE	Cat# 8220-09; SouthernBiotech, birmingham, AL, USA
Mouse anti-chicken TCRγδ biotin	Cat# 8230-08; SouthernBiotech, birmingham, AL, USA
Streptavidin PE-Cy7	Cat# 557598; BD pharmingen, san jose, CA, USA
Rabbit anti-chicken IFNγ	Cat# AHP945Z; bio-rad; hercules, CA, USA
Goat anti-rabbit IgG AF647	Cat# 4050-31; SouthernBiotech, birmingham, AL, USA

### RNA Isolation and Quantitative Real-Time PCR (qRT-PCR)

The mRNA expression of chicken TLRs and cytokine in cecal tonsils immune cells was performed as described previously (5). Briefly, total RNA from the cecal tonsils was extracted using a TRIzol reagent (Molecular Research Center, Cincinnati, OH, USA) following the manufacturer's instructions. The purity of RNA was determined by measuring absorbance in a NanoDrop spectrophotometer (Thermo Scientific, Waltham, MA, USA) at 260 and 280 nm. The cDNA synthesis was achieved via reverse transcription using 2 ng RNA template in a 20 μl reaction volume containing reaction buffer, 10 mM DTT, 0.5 mM dNTPs, 0.5 μg of oligo (dT) 15 primer, 8 units of RNAsin and 100 units of M-MLV reverse transcriptase (Promega, Madison, WI, USA) at 40°C for 1 h, followed with 95°C for 10 min. The mRNA expression was quantified by SYBR green method using 7,500 Real-Time PCR System spectrofluorometric thermocycler (Applied Biosystems, Waltham, MA, USA). The reaction mixture consisted of 10 μl PerfeCTa SYBR Green SuperMix (Quantabio, Beverly, MA, USA), 1 μl cDNA (0.1 ng) template, 5 μM of each primer and made up to 20 μl with RNAse-free water. The amplification protocol includes an initial denaturation of 95°C for 5 min (1 cycle), followed by 95°C for 20 s and 58°C for 45 s (40 cycles). Primers and annealing temperatures are listed in Table 3. The specificity of qRT-PCR product was verified through the melting curve generation at the end of each qRT-PCR run. The β-actin was used as the reference gene for normalization of Ct values. All data were normalized to the mRNA level of the mock group and reported as the fold-change (2^−ΔΔ^Ct method).

**Table 3:** Primers used in qRT-PCR for quantification of TLRs and cytokines mRNA expression.

**Primers**	**Oligonucleotides (5^′^−3^′^)**	**Annealing temperature**
β-actin	Forward: ACCGGACTATTACCAACACC Reverse: GACTGCTGCTGACACCTTCA	56°C
TLR 2	Forward: GCTCAACAGCTTCTCCAAGG Reverse: CCACCAGGATGAGGATGAAC	57°C
TLR 4	Forward: GGATGGACCGCAGCATGTTC Reverse: CAACCTGAGCAGCCTGTACG	56°C
IL-4	Forward: GGAGAGCATCCGGATAGTGA Reverse: TGACGCATGTTGAGGAAGAG	54°C
IL-10	Forward: CATGCTGCTGGGCCTGAA Reverse: CGTCTCCTTGATCTGCTTGATG	57°C

### Statistical Analysis

Statistical analyses were performed using the GraphPad Prism software (GraphPad Software version 8, Inc., La Jolla, CA, USA). Data were presented as mean ± standard error of mean (SEM) from 5 or 6 chickens in each group. Shapiro-Wilk test was performed to determine the normality of data. If data were normal distributed, then the difference between the groups were determined by analysis of variance (ANOVA) with Tukey's test, otherwise, Kruskal-Wallis test with Dunn's *post-hoc* test for multiple comparisons was performed. *P* < 0.05 was defined as statistically significant.

## Results

### Post-vaccination Pre-challenge Antibody Response Against *Salmonella* Antigens

**Figure 1:**
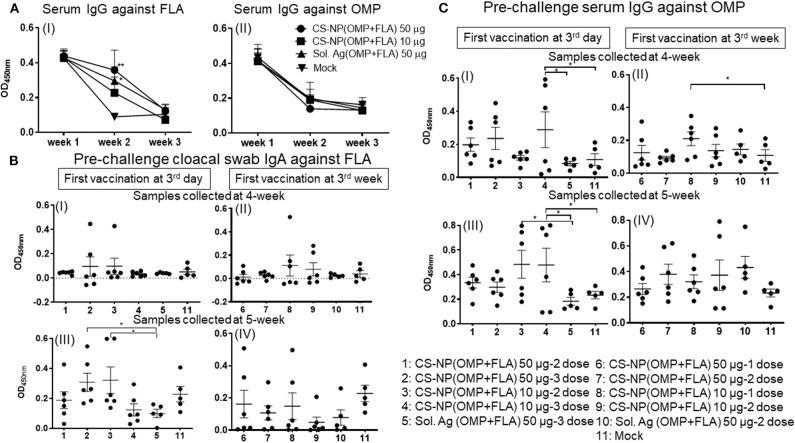
*Salmonella* specific antibody response post-vaccination and before challenge infection. Broiler birds were vaccinated with CS-NP(OMP+FLA) orally beginning at age 3-day or 3-week, with each dose containing either 10 or 50 μg OMP+FLA. Control birds received 50 μg of Sol.Ag(OMP+FLA). Cloacal swab and blood samples collected at different time points post-vaccination were analyzed for detection of specific IgA or IgG antibodies by ELISA. **(A)** Serum samples collected at age 3-day (week 1, before 1st vaccination), week 2 and week 3 were analyzed for IgG response against (I) FLA and (II) OMP. Significant difference between vaccinated and mock groups was analyzed by two-way ANOVA followed by Tukey *post-hoc* test. **(B)** Birds received 1st dose of vaccine at 3rd day (I and III) or at 3rd week (II and IV). Cloacal swabs collected at age 4-week (I and II) and 5-week before SE challenge infection (III and IV) were analyzed for IgA antibody response against FLA protein. **(C)** Serum samples collected on the same days as described in (B, I–IV) were analyzed for IgG antibody response against OMP. Significant difference between the indicated two groups was determined by one-way ANOVA followed by Tukey *post-hoc* test. *P < 0.05, **P < 0.01. Data were presented as mean ± SEM of 6 or 5 birds.

A day-old broiler chicks procured from a commercial source were from parental stocks vaccinated against *Salmonella*. To monitor the level of maternal antibodies and their interference with the first dose of CS-NP(OMP+FLA) vaccine, we analyzed OMP and FLA specific IgG antibody response in serum. The data showed high levels of FLA (but not OMP) specific antibodies at 2 weeks post-vaccination, but it reduced to basal level by 3 weeks (Figure 1A I & II).

Birds vaccinated at day 3 and week 3 (Table 1) were analyzed separately for cloacal swab IgA (Figure 1B) and serum IgG (Figure 1C) responses using samples collected at weeks 4 and 5. Interestingly, among all the vaccinated groups, FLA-specific IgA response at week 5 in cloacal swabs and OMP specific IgG at both week 4 and 5 were strongly induced (Figures 1B,C). While IgA response against OMP and IgG response against FLA were low, and when compared to control Sol.Ag and mock groups, values were not statistically significant (*P* > 0.05) (data not shown). When antibody responses in relation to the age of birds were compared, birds received the first dose at 3-day of age had higher levels of both specific IgA and IgG responses (Figures 1B,C). Comparing the data of IgA titers between samples collected at week 4 and 5, the levels were significantly (*P* < 0.05) enhanced in birds at week 5 received first dose at 3 day of age (Figure 1B I & III,) compared to at age 3 week first vaccination (Figure 1B II & IV). At 4-weeks of age, the IgG antibody response in birds that received 3 doses of vaccine were significantly higher (*P* < 0.05) vs. controls (Figure 1C I). In contrast, 3-day vaccinates with lower dose, when sampled at 5 weeks of age, induction of higher IgG response compared to Sol.Ag(OMP+FLA) and mock groups was observed (Figure 1C III). However, in birds that received vaccine at 3 weeks of age, the IgG response remained low (Figure 1C II & IV). Interestingly, compared to 50 μg/dose, in general, better antibody response was observed in birds given the 10 μg vaccine dose and it was further boosted after the third dose (Figures 1B,C).

### Post-challenge Antibody Response Against *Salmonella* Antigens

**Figure 2:**
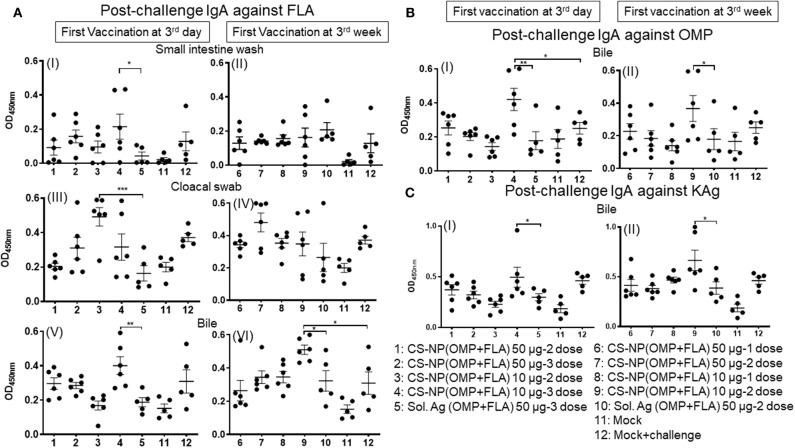
Specific IgA response in vaccinated birds post-challenge infection. Broilers were vaccinated as described in Fig legend 1, challenged at age 5-week, and 10 days later euthanized and samples collected for specific IgA antibody analysis by ELISA: **(A)** (I and II) Small intestine wash, (III and IV) Cloacal swab, (V and VI) Bile against FLA protein; **(B)** response against OMP in Bile (I and II); and **(C)** response against KAg in Bile (I and II). Data were presented as mean ± SEM of 6 or 5 birds. Significant difference between the indicated two groups were determined by one-way ANOVA followed by Tukey *post-hoc* test. *P < 0.05, **P < 0.01, ***P < 0.001.

**Figure 3:**
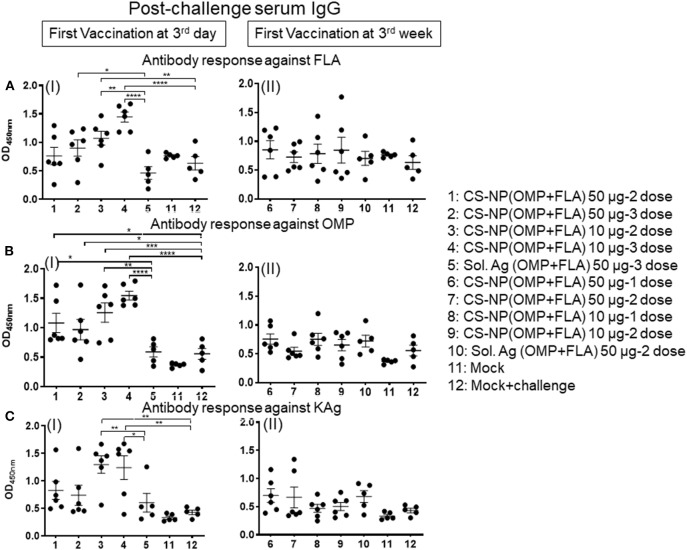
Specific IgG response in vaccinated birds post-challenge infection. Broilers were vaccinated as described in Fig legend 1, challenged at age 5-week, 10 days later euthanized and serum samples collected were analyzed for specific IgG antibody response by ELISA: against **(A)** FLA protein (I and II), **(B)** OMP (I and II), and **(C)** KAg (I and II). Data were presented as mean ± SEM of 6 or 5 birds. Significant difference between the indicated two groups were determined by one-way ANOVA followed by Tukey *post-hoc* test. *P < 0.05, **P < 0.01, ***P < 0.001, ****P < 0.0001.

Specific IgA responses in vaccinated birds after SE challenge infection were analyzed in small intestine washes, bile and cloacal swab samples collected on the day of necropsy (Figure 2). Birds received 2 or 3 doses of 10 μg/dose of CS-NP(OMP+FLA) vaccine had higher FLA-specific IgA response compared to the Sol.Ag group (Figure 2A). In small intestine washes and bile, 3 day vaccinates given the 10 μg × 3 doses, a robust FLA, OMP and KAg specific IgA response was seen (Figures 2AI,V,BI,CI). This was especially notable in the bile where significantly (*P* < 0.05) higher amounts of antigen-specific IgA antibodies were secreted compared to control groups (Figures 2A,V,VI, B). Birds that received two doses of vaccine (first dose at 3 days of age) had higher FLA-specific IgA response in cloacal swabs than controls (Figure 2A III). Serum IgG levels in early vaccinates had significantly (*P* < 0.05–*P* < 0.0001) higher IgG levels for FLA, OMP, and KAg when compared to latter vaccinates (Figure 3). Moreover, birds that received 10 μg/dose of CS-NP(OMP+FLA) had a higher specific IgG response compared to 50 μg/dose group (Figures 3AI, BI, CI).

### Challenge Bacterial Load in the Cecum of Vaccinated Birds

**Figure 4:**
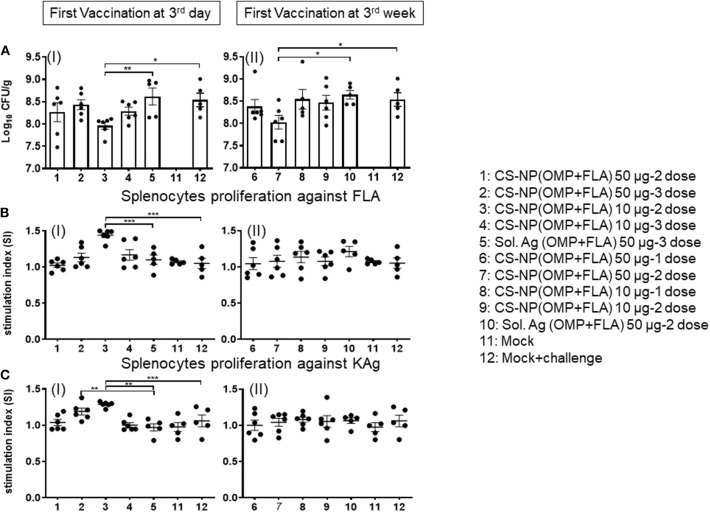
SE load in the cecal content of vaccinated and challenge birds, and *Salmonella* specific lymphocytes proliferation response in splenocytes. **(A)** *Salmonella* CFU was enumerated in cecal content by plating on XLD agar plates (I and II). **(B,C)** Splenocytes harvested on the day of necropsy were stimulated with **(B)** FLA protein (I and II) or **(C)** KAg (I and II) for 48 h and lymphocytes stimulation index (SI) value was calculated by the mean OD of specific antigen (FLA/KAg) stimulated proliferation/mean OD of non-stimulated proliferation by a colorimetric assay. Each bar is the mean ± SEM of 6 or 5 birds. Significant differences were calculated between vaccinated and Sol. Ag (OMP+FLA) or mock group by one-way ANOVA followed by Tukey *post-hoc* test. *P < 0.05, **P < 0.01, ***P < 0.001.

All the vaccinated birds except a mock control group were challenged with SE, 5 × 10^8^ CFU/bird. In uninfected mock control, SE CFUs were not detected (Figure 4A I & II). Among all the CS-NP(OMP+FLA) vaccinates, those that received early vaccination with 2 doses of 10 μg/dose had the lowest SE in cecal contents (log_10_ 7.96 CFU/g); these data were statistically significant (*P* < 0.05) compared to mock challenge group (log_10_ 8.54 CFU/g) and Sol.Ag(OMP+FLA) group (log_10_ 8.62 CFU/g) (*P* < 0.01) (Figure 4C I). Birds that received the first dose of vaccine at 3 weeks of age (2 doses of 50 μg/dose) had SE counts (log_10_ 8.03 CFU/g) significantly lower (*P* < 0.05) than control groups (Figure 4A II).

### *Salmonella* Antigen Specific Lymphocyte Proliferation in Splenocytes From CS-NP(OMP+FLA) Vaccinates

The FLA antigen-specific lymphocyte proliferative response was significantly increased (*P* < 0.001) in birds that received first dose of CS-NP(OMP+FLA) at 3 days of age and subsequent doses (10 μg/dose) compared to mock and Sol.Ag groups (Figure 4B I). The KAg-specific proliferative response was significantly increased (*P* < 0.01) in both CS-NP(OMP+FLA) vaccine (three doses of 10 μg/dose; two doses 50 μg/dose) compared to Sol.Ag group (Figure 4C I). In addition, 2 doses of 10 μg/dose CS-NP(OMP+FLA) early vaccinates, KAg-specific proliferative response was significantly increased (*P* < 0.001) compared to mock challenge group (Figure 4C I). However, birds that received first dose of vaccine at three weeks of age did not have any increase in specific lymphocytes proliferation response (Figures 4BII, CII). The OMP-specific splenocyte response was not statistically different in any of the vaccinated groups (data not shown).

### *Salmonella* OMP+FLA-Antigen Specific IFNγ Production in Lymphocyte Subsets of CS-NP(OMP+FLA) Vaccinates

**Figure 5:**
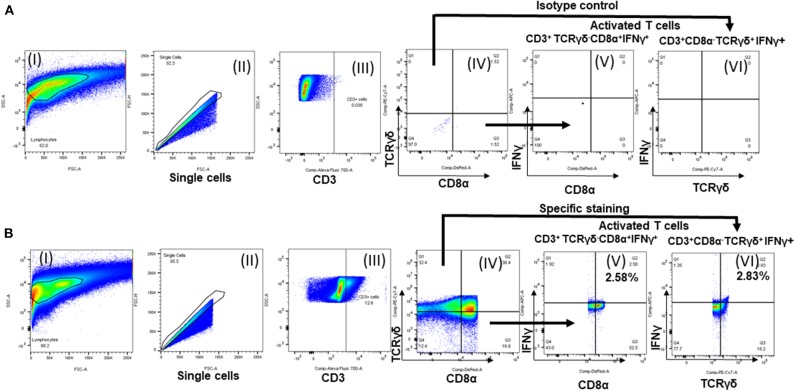
Flow cytometry analysis of splenocytes of vaccinated and challenge birds. Lymphocytes gating strategy followed to show the percentage of specific activated lymphocyte subsets. **(A)** Isotype control and **(B)** specific antibody immunostained splenocytes analysis. (I) Lymphocytes gating; (II) single cells selection; (III) cells gated initially for CD3; (IV) subdivided the CD3^+^ population into TCRγδ^+^/TCRγδ^−^ and CD8α^+^/ CD8α^−^; IFNγ^+^ secreting (V) TCRγδ^−^CD8α^+^ cells and (VI) TCRγδ^+^CD8α^−^ cells.

**Figure 6:**
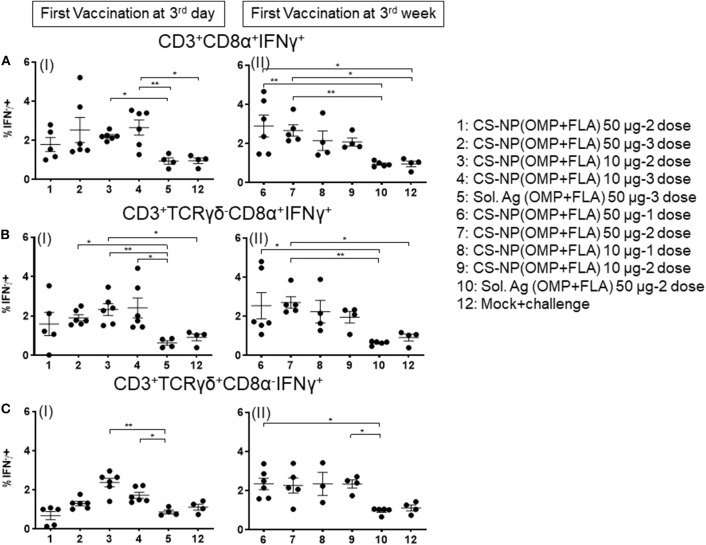
*Salmonella* antigen specific proliferated T lymphocyte subsets in the splenocyte of vaccinated and SE challenge birds. Splenocytes isolated on the day of necropsy were restimulated with vaccine antigens (OMP+FLA). The frequency of activated IFNγ^+^ secreting lymphocytes were determined by flow cytometry. Average frequency of lymphocytes: **(A)** CD3^+^CD8α^+^IFNγ^+^ (I and II); **(B)** CD3^+^TCRγδ^−^CD8α^+^IFNγ^+^ (I and II); and **(C)** CD3^+^TCRγδ^+^CD8α^−^IFNγ^+^ (I and II) from all the experimental groups were quantified. Data were presented as average percentage of indicated lymphocyte subset ± SEM. Significant differences were calculated between vaccinated and Sol. Ag (OMP+FLA) or mock challenge group by Kruskal–Wallis test followed by Dunn's multiple comparison test. *P < 0.05, **P < 0.01.

Chicken IFNγ is a pivotal cytokine for pathogen clearance in the host immune defense response, and has been regarded as a reliable indicator to assess cell mediated immunity in chickens (14, 15). Based on the expression of combination of specific phenotypic markers on surface of chicken lymphocytes, cells were grouped as cytotoxic T cells (CTLs)/γδ T cells (CD3^+^CD8α^+^) and exclusive CTLs (CD3^+^TCRγδ^−^ CD8α^+^) based upon phenotype-specific cell markers indicated in parenthesis. A representative gating strategy of lymphocytes secreting IFNγ is shown in Figure 5. In splenocytes of 10 μg/dose CS-NP(OMP+FLA) early vaccinated (at 3rd day) birds (both 2 or 3 doses), stimulated with pooled SE antigens (OMP+FLA) observed significantly higher (*P* < 0.05 to 0.01) IFNγ^+^ secreting CTLs/γδ T cells compared to Sol.Ag (OMP+FLA) group (Figure 6A I). In all the 50 μg/dose CS-NP(OMP+FLA) vaccinates received the prime dose at 3rd week, IFNγ^+^ secreting CTLs/γδ T cells were significantly higher (P < 0.05 to 0.01) compared to mock challenge and Sol.Ag (OMP+FLA) groups (Figure 6A II). Among them, specifically detected an increased frequency of IFNγ producing CTLs (CD3^+^TCRγδ^−^CD8α^+^IFNγ^+^) in all the CS-NP(OMP+FLA) vaccinated birds (Figure 6B I & II). Among the early vaccinated birds given 2 doses of 10 μg/dose of CS-NP(OMP+FLA) vaccine, significantly increased frequency of IFNγ producing CTLs (*P* < 0.05 to 0.01) compared to both mock challenge and Sol.Ag (OMP+FLA) groups were detected (Figure 6B I). In late vaccinated birds, 2 doses of 50 μg/dose of CS-NP(OMP+FLA) vaccine was required to achieve a higher level of IFNγ producing CTLs frequency (Figure 6B II). In addition, in early 10 μg/dose CS-NP(OMP+FLA) vaccinated birds, IFNγ producing γδ T cells (CD3^+^TCRγδ^+^CD8α^−^IFNγ^+^) were augmented compared to the Sol.Ag (OMP+FLA) group (Figure 6C I). In late vaccinated groups, received one dose of 50 μg/dose and 2 doses of 10 μg/dose observed higher frequency of IFNγ producing γδ T cells compared to Sol.Ag (OMP+FLA) group (Figure 6C II).

### CS-NP(OMP+FLA) Vaccine Induced TLRs and Cytokine mRNA Expression in Cecal Immune Cells

**Figure 7:**
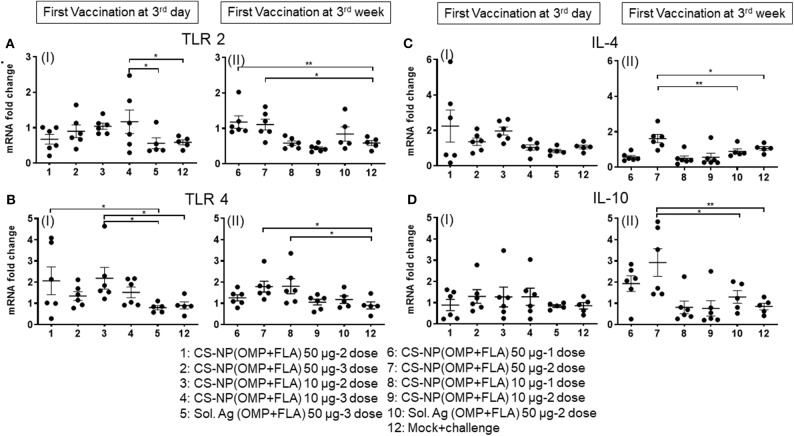
TLR and cytokine gene expression in vaccinated and SE challenge birds. Cecal tonsils harvested on the day of necropsy were analyzed for the expression of TLRs and cytokine mRNA in CS-NP(OMP+FLA) vaccinated birds by qRT-PCR. **(A)** TLR 2 (I and II), **(B)** TLR 4 (I and II), **(C)** IL-4 (I and II) and **(D)** IL-10 (I and II). Fold-change in gene expression was calculated after correcting for β-actin mRNA value and normalizing to mRNA content of mock group. Data were presented as mean ± SEM of 6 or 5 birds. Significant difference between the indicated two groups were determined by one-way ANOVA followed by Tukey *post-hoc* test. *P < 0.05, **P < 0.01.

Extracted total RNA from the cecal tonsils of birds were analyzed for the expression of different TLRs and cytokine mRNA by quantitative qRT-PCR. Our results identified the increased expression of TLRs 2 and 4 in CS-NP(OMP+FLA) vaccinated birds (Figures 7A,B). Among the early vaccinated birds given 3 doses of 10 μg/dose of CS-NP(OMP+FLA) a significantly (*P* < 0.05) increased level of TLR 2 mRNA compared to both mock and Sol.Ag(OMP+FLA) groups was observed (Figure 7A I). Early vaccinated birds received 10 μg/dose 2 doses of CS-NP(OMP+FLA) vaccine, a significantly higher (*P* < 0.05) TLR 4 mRNA expression compared to both mock challenge and Sol.Ag(OMP+FLA) groups was detected (Figure 7B I). Birds received 50 μg/dose 2 doses of CS-NP(OMP+FLA) vaccine had significantly higher (*P* < 0.05) TLR 4 mRNA expression than Sol.Ag(OMP+FLA) group (Figure 7B I). In the cecal tonsils of birds vaccinated at 3-weeks of age with 50 μg/dose after both 1 or 2 doses vaccination, significantly higher (*P* < 0.05) TLR 2 mRNA expression was observed compared to mock group (Figure 7A II). Birds received the first dose of CS-NP(OMP+FLA) vaccine at 3-week of age with 1 dose of 10 μg/dose and 2 doses of 50 μg/dose had significantly higher (*P* < 0.05) TLR 4 mRNA level compared to mock group (Figure 7B II). Furthermore, the cecal tonsils of birds vaccinated first dose at 3 weeks (but not 3 day) of age with 2 dose of 50 μg/dose of CS-NP(OMP+FLA) had significantly (*P* < 0.05) elevated IL-4 and IL-10 mRNA levels compared to mock and Sol.Ag(OMP+FLA) groups (Figures 7C,D).

## Discussion

To develop a better vaccination strategy against SE colonization and shedding in the chicken industry, it is important to understand vaccine-induced immune mechanisms following challenge infection. In broilers administrated orally with CS-NP(OMP+FLA) vaccine, we examined both the age- and dose-dependent cellular and humoral immune responses along with vaccine efficacy in a bacterial challenge trial. We observed reduced bacterial load in the ceca of birds that received 2 doses of 10 μg/dose beginning at 3 days of age and 50 μg/dose at 3 weeks of age, suggesting the need of a second (booster) vaccination. As well, the benefits of vaccination at younger age were documented. Subunit antigen-based *Salmonella* vaccination for control of disease in poultry provides poor immune protection (16). In contrast, the bacterial challenge trial results in this study suggest that early vaccination using low amounts (10 μg/dose) of subunit antigens OMP and FLA when delivered in CS-NP have the potential in inducing a protective immune response. Induction of high levels of antigen-specific serum IgG and cloacal and intestinal IgA secretion in birds vaccinated at 3 days of age, after both vaccination and challenge infection were evident. Additionally, cell mediated immune response as measured by both antigen specific lymphocyte proliferation and IFNγ-producing T cells were increased in the splenocytes of CS-NP(OMP+FLA) vaccinates. Although three doses of 10 μg/dose in early vaccinates induced higher antibody and cell mediated immune response after challenge infection, it was not resulted in reduced SE load >2 doses early vaccinates.

The presence of specific IgA secretion in bile indicates specific antibody response in the gut, but it did not result in immune protection from subsequent SE challenge (17). An increased bile IgA response in bile along with augmented antibody response in small intestinal wash and cloacal swab and IgG in serum; specific activation of IFNγ secreting lymphocytes together indicate induction of both mucosal and systemic immunity induced by CS-NP(OMP+FLA) vaccine in early vaccinates. To note, oral vaccination was not interfered by the presence of maternal antibodies. The IFNγ producing T cells and other lymphocytes and cytokines are an important component of immune protection for intracellular pathogens (13, 18). This study demonstrates that the nanoparticle as a vector in the form of CS-NP(OMP+FLA) vaccinations induced prominent FLA-specific lymphocyte proliferation and increased frequency of different IFNγ producing T cells subsets.

In mice, the role of cell mediated immunity is associated with protection against *Salmonella* burden (19). In chickens, when live and killed *Salmonella* vaccines comparative performance data was analyzed, FLA-specific splenocyte proliferation correlated to SE shedding in live (but not killed) vaccinates. This effect was associated with increased CD3^+^ lymphocytes population, suggesting that protection against SE infection is augmented by cell mediated immunity (20). Consistent with this data, FLA- and KAg-specific lymphocytes proliferation response was increased in birds received CS-NP(OMP+FLA) vaccine at 3 days of age. Activated (IFNγ^+^ secreting) CTLs and γδ T cells were increased in CS-NP(OMP+FLA) vaccinated birds irrespective of the first dose of the vaccine was administered at 3 days or 3 weeks of age.

High levels of TLR 2, TLR 4, IL-4, and IL-10 gene expression were detected in the cecal tonsils of birds received CS-NP(OMP+FLA) vaccine. TLR 2 and TLR 4 are associated with pro-inflammatory cytokine production in chickens (21), while cytokines IL-4 and IL-10 typically play a role as anti-inflammatory cytokines which inhibit the inflammatory response (22, 23). Cytokine IL-10 is an essential mediator of inhibitory functions of B cells (24). This type of cytokine response is associated with enhanced mucosal and systemic antibody response, suggesting the induction of both pro-inflammatory and anti-inflammatory responses in CS-NP(OMP+FLA) vaccinates. Humoral immunity contributes to SE clearance during recurrent infection (25). Considering the TLRs are associated with non-specific innate responses and that do not confer direct protection, recognition of TLRs triggers functional maturation of dendritic cells and leads to initiation of antigen-specific adaptive immune responses (26). This innate to specific adaptive immune transition in CS-NP(OMP+FLA) vaccinates occurred in early vaccinates.

In this study, irrespective of first dose of CS-NP(OMP+FLA) vaccine administered early or latter, the candidate vaccine shown its ability to elicit immune response against challenge infection. When compared to 50 μg/dose of Sol.Ag(OMP+FLA) inoculated birds, all CS-NP(OMP+FLA) vaccinates showed increased immune response after challenge infection, suggesting the enhanced efficiency of chitosan nanoparticle as a vaccine vector. The fact is that all these elevated humoral and cell mediated immune response indices using low dose of vaccine resulted in reduction in SE load of 0.7 Log_10_ CFU/g of cecal contents. Though this indicates the potential of CS-NP(OMP+FLA) vaccine as a viable candidate in broilers, further improvements are required in its formulation by incorporating additional adjuvants and other *Salmonella* serotype antigens to widen the breadth of immunity. Also, it is important to include commercial live *Salmonella* vaccine as a control for comparative analysis.

## Conclusion

In conclusion, oral delivered CS-NP(OMP+FLA) *Salmonella* subunit vaccine has the capacity of inducing both cell mediated and humoral immune response in broilers. For stronger and long-term protection, it is highly recommended that broilers should be given optimal dose of vaccine antigens in CS-NP, and the first dose should be given at 3rd day age or within first week after hatch followed by a booster after 2 weeks. Early vaccination is important because SE infection of young chicks results in high levels of environmental contamination and rapid transmission of pathogens. Further studies are required to improve the candidate vaccine's efficacy and performance through modifications in the formulation and incorporating other *Salmonella* serotype antigens, and dynamic analysis of vaccine protection efficiency in challenge trials.

## Data Availability Statement

The datasets generated for this study are available on request to the corresponding author.

## Ethics Statement

Our study was in accordance with the recommendations of Public Health Service Policy, United States Department of Agriculture Regulations, the National Research Council's Guide for the Care and Use of Laboratory Animals, and the Federation of Animal Science Societies' Guide for the Care and Use of Agriculture Animals in Agricultural Research and Teaching. We followed all the relevant institutional, state, and federal regulations and policies regarding animal care and use at The Ohio State University. Chickens were maintained, euthanized and samples collected in accordance with the approved protocol of the Institutional Animal Care and Use Committee at The Ohio State University (Protocol number 2016A00000060).

## Author Contributions

YH, SR, and GR conceived and designed the project. YH and GR composed the manuscript. YH performed the experiments and analyzed the data. SR synthesized CS-NP vaccines. Vaccination and challenge trial in chickens, sample collection, and laboratory experiments were supported by SR, NF-R, JS, and VP. VP performed the flow cytometry data analyses. All authors provided critical feedback on the manuscript prior to publication and have agreed to the final content.

## Conflict of Interest

The authors declare that the research was conducted in the absence of any commercial or financial relationships that could be construed as a potential conflict of interest.
